# The P2 purinoceptors in prostate cancer

**DOI:** 10.1007/s11302-022-09874-2

**Published:** 2022-06-30

**Authors:** Zilin Wang, Sha Zhu, Sirui Tan, Yuhao Zeng, Hao Zeng

**Affiliations:** 1 The Department of Urology, Institute of Urology, West China Hospital, Sichuan University, Chengdu, China; 2 Department of Abdominal Cancer, Medical School, West China Hospital, Sichuan University, Cancer Center, Chengdu, West China China

**Keywords:** P2 purinoceptors, Prostate cancer, P2XR, P2YR, Akt, ERK1/2

## Abstract

P2 purinoceptors are composed of ligand-gated ion channel type (P2X receptor) and G protein-coupled metabolite type (P2Y receptor). Both these receptors have played important roles in the prostate cancer microenvironment in recent years. P2X and P2Y receptors can contribute to prostate cancer’s growth and invasiveness. However, the comprehensive mechanisms have yet to be identified. By summarizing the relevant studies, we believe that P2X and P2Y receptors play a dual role in cancer cell growth depending on the prostate cancer microenvironment and different downstream signalling pathways. We also summarized how different signalling pathways contribute to tumor invasiveness and metastasis through P2X and P2Y receptors, focusing on understanding the specific mechanisms led by P2X4, P2X7, and P2Y2. Statins may reduce and prevent tumor progression through P2X7 so that P2X purinergic receptors may have clinical implications in the management of prostate cancer. Furthermore, P2X7 receptors can aid in the early detection of prostate cancer. We hope that this review will provide new insights for future mechanistic and clinical investigations into the role of P2 purinergic receptors in prostate cancer.

## Introduction

One of the most common and lethal solid tumors in males is prostate cancer [1]. Most patients are diagnosed with hormone-sensitive prostate cancer (HSPC). Although androgen deprivation therapy (ADT) is initially effective, tumors continually produce autocrine or paracrine androgens, resulting in castration-resistant prostate cancer (CRPC) [1–3]. Local therapies (radical prostatectomy and radical radiotherapy) could cure early-stage localized prostate cancer [4]. Unfortunately, despite developing more novel and effective strategies in recent years, the prognosis of metastatic prostate cancer remains poor [5]. There is the utmost need to establish the alternative therapies. Synthetic lethality, immunotherapy, and other targetable molecular aberrations have recently been attempted for metastatic prostate cancer. Still, each of them has limitations [6].

Purinergic receptors bind with various purine nucleotides and nucleosides, such as ATP, ADP, AMP, and adenosine and are essential in the cancer microenvironment [7]. These receptors are classified as P1 (adenosine receptors) and P2 (receptors for ATP and analogues). P2 purinoceptors are further classified as ligand-gated ion channels (P2X) and G protein-coupled metabolites (P2Y and other types) [8]. For P2XR, the ligand-gated ion channel, alterations in ion channel expression, and tumor activity regulate tumor cell proliferation and apoptosis. Tumor angiogenesis is regulated by K^+^ and Ca^2+^ ion channels, while tumor invasion and metastasis are regulated by Na^+^ and Cl^−^ ion channels [7]. In the meantime, G protein-coupled receptors (GPCRs) are collectively referred to as peptide membrane protein receptors [9, 10]. G protein-coupled receptors are important in metastatic cancer progression and are considered promising targets for cancer treatment [11].

P2 receptors currently play a vital role in the tumor microenvironment. P2 receptor subtype activation or inhibition in the tumor microenvironment can result in cancer cell death or growth inhibition. Meanwhile, P2 receptor stimulation or agonist frequently results in an inflammatory response, which contributes to immune stimulation while also activating various immunosuppressive mechanisms, particularly in the tumor microenvironment [12–15]. P2 receptors are found in cervical, gastrointestinal, liver carcinoma, and prostate cancer cells and can activate esophageal cancer cell proliferation via the extracellular signal-regulated kinase 1/2 (ERK1/2) pathway [16–22]. The role of P2 receptors in prostate cancer has been underappreciated; thus, this review aims to summarize current knowledge in this area.

## P2XRs and their role in prostate cancer

**Fig. 1:**
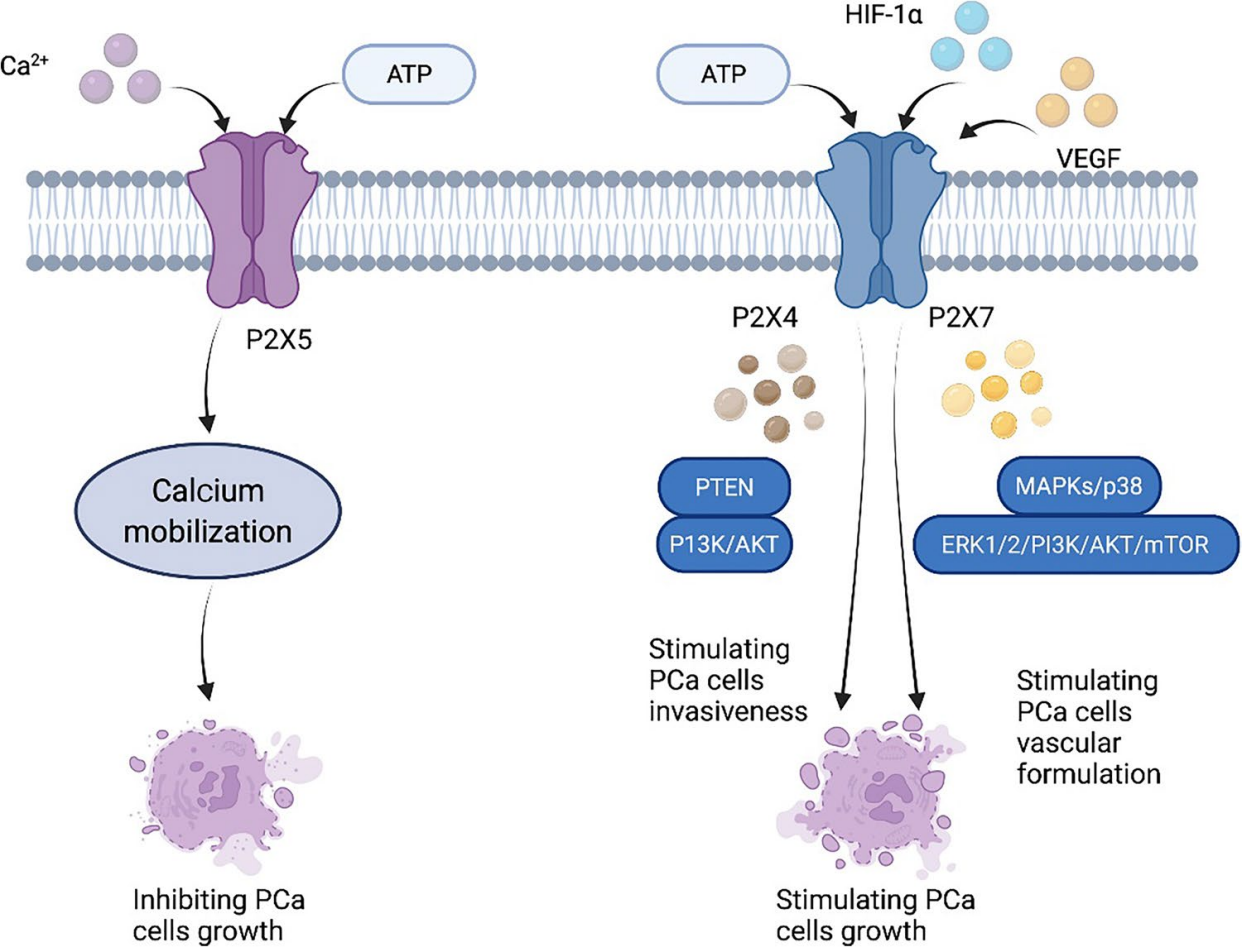
The pathway of P2XR in prostate cancer. When initiating the feedforward cycle of ATP release and P2X4 and P2X7, it increases intracellular Ca^2+^ and triggers the involvement of MAPKs, p38, ERK1/2, and PI3K. Meanwhile, the PI3K-AKT-mTOR pathway and multiple interacting cellular signaling cascades, especially the complex crosstalk between MAPK and AR, can further promote growth and metastasis in prostate cancer cells. In contrast to growth, ATP can inhibit CRPC cell growth by 90% through a rapid, transient increase in cytoplasmic free Ca^2+^ by P2X5, inducing apoptosis in a Ca.^2+^-independent mechanism

Previous research has revealed that P2X7 receptors are expressed in various human cancers [22]. Recent studies have found an exosome/microvesicle and miRNA-dependent mechanism of P2X7 receptor having prometastatic activity in metastatic cancer [23, 24]. P2X7 expression appears to be required for tumor growth in several cancer types, including mesothelioma [25] and melanoma [26, 27]. P2X receptors (P2XRs) are ATP-gated ion channels that are made up of seven distinct subunits (P2X1-7) [28], which form a chalice-shaped tribe. Each subunit comprises a large ectodomain that contains one ATP binding site, intracellular N and C terminals, and two transmembrane domains [29]. They have a diverse distribution and functional characteristics in vivo [30]. According to the growing evidence, P2XRs play various roles in malignant tumors. P2XRs are involved in tumor cell proliferation, differentiation, angiogenesis, biogenetics, metastasis, cancer-related pain, and tumor immunity [30–32] (Fig. 1).

### P2XR in prostate cancer cell growth

P2XRs that are liganded/activated by ATP, such as P2X4, P2X5, and P2X7, are expressed at various stages of prostate cancer, including localized prostate cancer, HSPC, and metastatic castration-resistant prostate cancer (mCRPC) [33–35]. P2X7 promotes prostate cancer cell growth by increasing Ca^2+^ influx or mitochondrial involvement. According to recent research, ATP released under hypotonic pressure promotes the growth of prostate cancer cells [36]. Hypotonic stress starts the feedforward cycle of ATP release and purinergic receptor signalling, which then increases intracellular Ca^2+^ and activates mitogen-activated protein kinases (MAPKs), p38, ERK1/2, and phosphatidylinositol 3-kinase (PI3K) [37]. In tumors derived from patients and prostate cancer cell lines, p38 MAPK is associated with cell proliferation. IL-6 influences prostate cancer cells via AMPK/SIRT1/p38 MAPK signalling, just as it does in neuroendocrine differentiation, p38 MAPK also regulates cell death in solid tumors in a dual manner depending on the stimulus and affected cell type [38]. In prostate cancer, active p38 promotes apoptosis, but this requires coordination with other signals, such as the PI3K/AKT pathway, to determine cellular outcomes. The PI3K-AKT-mTOR pathway and multiple interacting cellular signalling cascades, particularly the complex association between MAPK and AR, can progress prostate cancer. ADT and other PI3K-AKT-mTOR-targeted therapies are being studied in the clinic [39].

Accumulation of hypoxia-inducible factor-1 alpha (HIF-1α) can modulate P2X7, increasing invasiveness and stimulating hypoxia tumor angiogenesis. Tumors with functional P2X7 expression can be detected in vascular endothelial growth factor (VEGF) enrichment and a dense vascular network [40]. Various studies demonstrate that downregulation of P2X7 reduces HIF-1α and VEGF levels and angiogenesis [40–42]. Intratumoral injection of Avastin (bevacizumab) to block VEGF or direct silencing of the P2X7 receptor in vivo can inhibit tumor cell growth and angiogenesis. However, in monocytes, brief and prolonged activation of P2X7 promotes VEGF release. P2X7/HIF-1α/VEGF promotes breast cancer cell invasion and migration [43]. Although there is no direct association between P2X7 and HIF-1α in prostate cancer, the interaction of VEGFR-2 and P2X7 gene polymorphisms is associated with better overall survival [35, 44].

Meanwhile, P2X4 increases the formation of prostate cancer and is a clinically targetable candidate for therapeutic targeting. P2X4 activation increases prostate cancer cell migration and invasion via epithelial-mesenchymal transition (EMT) in prostate cancer cells [45]. Furthermore, higher P2X4 protein expression was observed in prostate cancer cells with PTEN loss and positive ERG protein expression [45]. P2X4 protein expression is linked to an increased risk of metastasis, and PTEN is a PI3K negative regulator that leads to AKT dephosphorylation [32, 54]. The active form of AKT is associated with a poor prognosis in various cancers and promotes mesenchymal-like properties and metastasis in prostate cancer cells [46]. P2X4 protein expression above the median, in conjunction with PTEN loss, is linked to an increased risk of metastasis and biochemical recurrence after prostatectomy [29, 32]. In vitro, blocking P2X4 purinoceptors reduced the viability of prostate cancer cells, implying potential implications for prostate cancer [32, 54].

In addition to growth, P2XR can cause prostate cancer cell apoptosis in CRPC. Prostate carcinoma cells express functional P2-purinergic receptors, which may inhibit the development of these cells by inducing apoptosis and increasing cellular differentiation [47]. Specifically, ATP can inhibit CRPC cell growth by 90% by inducing apoptosis in a Ca^2+^−independent manner via a rapid, transient increase in cytoplasmic free Ca^2+^ by P2X5. This means that ATP and adenosine may have a high potential for effectively inhibiting the growth of androgen-sensitive, androgen-independent, and bone-adaptive stages of prostate cancer [48, 49]. Although the mechanism of ATP and adenosine on prostate cancer growth inhibition has not been determined, these inhibitory effects may be due to one or more of these receptors.

### P2XR in prostate cancer cell invasiveness

P2X7 is associated with ATP-stimulated cancer invasiveness in many cancers, including prostate cancer. In prostate cancer, the non-functional P2X7 allele (rs3751143) is less aggressive than the functional one [50]. P2X7 promotes the invasive growth of prostate cancer through PI3K/Akt signaling pathway, ERK1/2 signalling pathway, and specific EMT/invasion-related genes [51]. Snail, IL-8, metalloproteinase (MMP)-3, E-cadherin, and claudin-1 are all EMT factors influenced by ATP and BzATP [50]. According to some studies, P2X7 depletion reduces the ATP-induced effects on these genes. The antagonist KN62, on the other hand, only inhibits ATP regulation of Snail, E-cadherin, and claudin-1. The antagonist KN62 reduces phosphorylation of the downstream targets GSK3β Ser9 and 70 S6K Thr386 to inhibit constitutive Akt levels. P2X7 knockdown significantly inhibited the phosphorylation of the PI3K/Akt and ERK 1/2 signalling pathways activated by ATP and BzATP [35]. LY294002 and U126, which inhibit PI3K/Akt and ERK1/2, respectively, reduce EMT. P2X7-mediated, ATP-driven invasiveness in PTEN-positive prostate cancer cells could be suppressed by atorvastatin [52, 53].

Hypoxia primarily promotes cancer invasion through two different mechanisms. HIF-1α activates P2X7 and the receptor for advantaged glycation end products (RAGE) expression. The expression of P2X7 and RAGE then mediates AKT and ERK1/2 phosphorylation and nuclear factor-kappa B (NF-ΚB) translocation [26]. On the other hand, HIF-1α affects tumor invasion and metastasis by increasing the activity of matrix metalloproteinases (MMPs), especially MMP-9, an endopeptidase that has been shown to degrade the extracellular matrix, promote tumor angiogenesis, and play an important role in tumor invasion and metastasis. P2X7, in general, acts as a RAGE promoter in the phosphorylation of AKT and ERK1/2 [54]. Extracellular ligands stimulate P2X7, causing phosphorylation of NF-ΚB nuclear translocation and, as a result, increased expression of MMP-9 and MMP-2, resulting in EMT, invasion, migration, and bone metastasis of prostate cancer cells [55, 56].

### The clinical implication of P2XR in prostate cancer

Statins may lower the risk of aggressive prostate cancer by counteracting the antagonistic effects of extracellular ATP via P2X7 [52]. Statins inhibit the aggressive growth of PTEN-positive cells induced by extracellular ATP by rapidly downregulating the nuclear level of phosphorylated Akt via P2X7. EHBP1, a protein, is involved in actin reorganization and is associated with an increased risk of prostate cancer in men with two T alleles at rs2710647. Its inhibition prevents nuclear pAkt depletion and the aggressive suppression caused by atorvastatin [30]. Furthermore, P-Rex1, a gene associated with prostate cancer invasive growth and metastasis, was found to be involved in atorvastatin-induced P2X7 signalling. They quickly translocate in response to P2X7 signaling and are then mediated by non-HMG-CoA reductase-dependent effects of statins. Because of the statin-related mevalonate effect, atorvastatin may prevent the invasive growth of prostate cancer in early lesions [52]. P2X7 isoform B also plays a role in tumors [57]. Because of its transforming activity, P2X7 isoform B has gradually gained attention for its role in cancer. Indeed, with evidence that this isoform promotes carcinogenesis in glioma and osteosarcoma [58, 59], P2X7 isoform B has been shown to activate the NFATc1 proliferation pathway, promoting soft agar invasion and ATP secretion [34]. Concurrently, it can withstand the effects of antitumor treatments such as chemotherapy or radiotherapy [57], but there is no information on this aspect of prostate cancer. In the future, we should focus on P2X7 isoform B’s role in the clinical treatment of prostate cancer.

P2XR aids in prostate cancer therapy, but it also aids in the early detection of prostate cancer. This stage-specific labelling pattern has been divided into three categories of purinergic receptor translocation (PRT0-3). PRT0 (no label), P2X1, P2X2, and P2X7 are not present in normal or benign hyperplastic prostatic epithelial tissue that will not develop into prostate cancer during the next five years [57]. They are then expressed in PRT1 (nuclear label). P2X1, P2X2, and P2X7 receptors move from the nucleus to the cytoplasm in PRT2 (cytoplasmic label). Only full-scale sampling revealed a tumor in the PRT2 stage. As cancer develops, the apical labelling of P2X1, P2X2, and P2X7 on the epithelium is referred to as PRT3, and cancer is typically diagnosed using H&E staining [58]. H&E staining of a small portion of the prostate reveals the majority of tumors. As a result, most cancer patient samples show no morphological abnormalities [59]. However, all of these cancer cases have P2X7 labelling features in all sampled cores. The radical prostatectomy section tagged with anti-P2X7 in an advanced prostate cancer case revealed PRT3 in all glandular acini. Because of this field effect, humans may detect prostate cancer from a single core. H&E staining showed that the prostate tissue’s PRT1 or PRT2 labelling pattern was normal or benign. As a result, it can be used to detect the early stages of potential tumor progression [34, 59].

## P2YRs and their role in prostate cancer

**Fig. 2:**
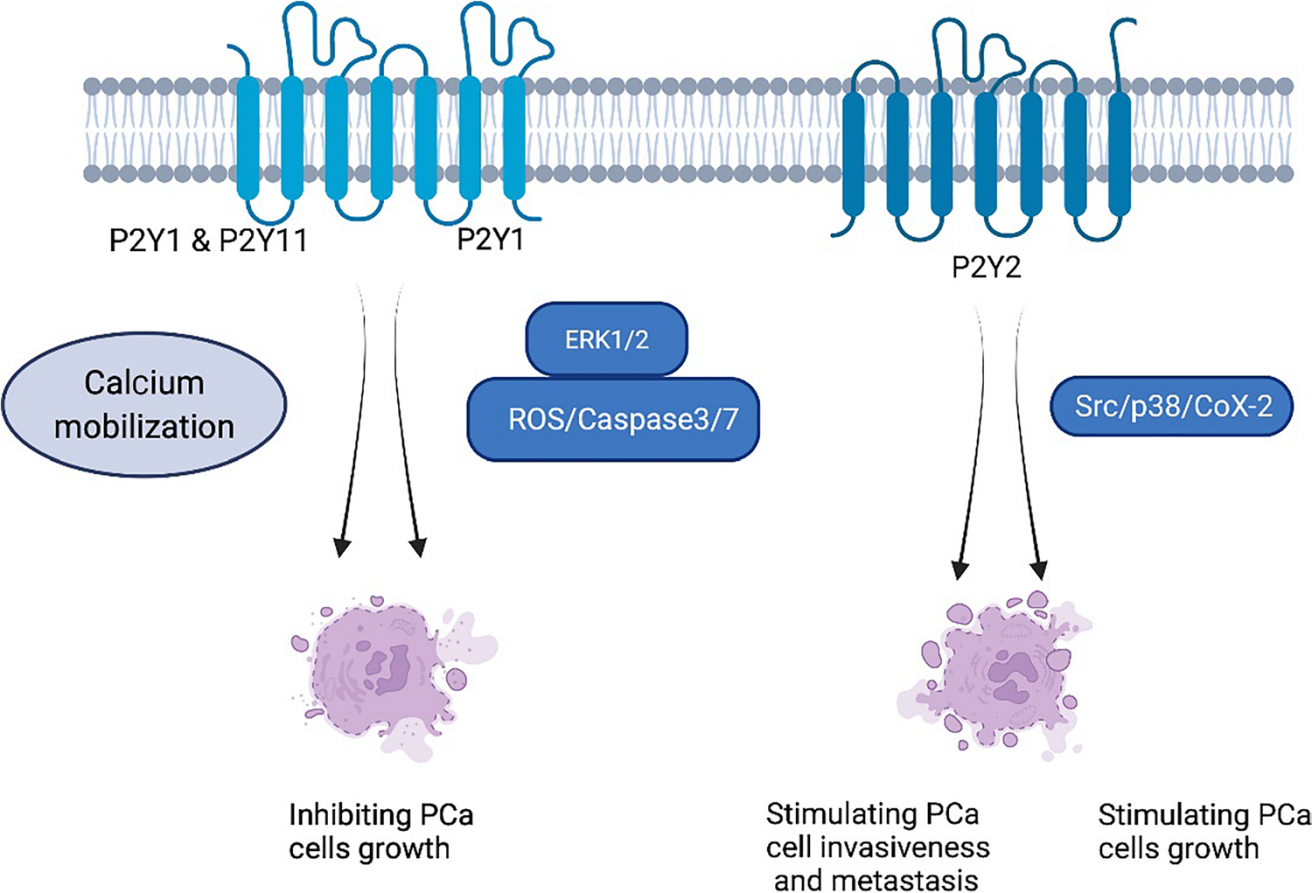
The pathway of P2YR in prostate cancer. The activation of P2Y2, in turn, activated Src, which phosphorylated p38 leading to COX-2 overexpression, causing resistance to apoptosis in prostate cancer cells. By contrast, P2Y1 and P2Y11 receptors induce phosphorylation of ERK1/2, and activation of P2Y1R induced apoptosis via the caspase 3/7 and reactive oxygen species (ROS) signaling pathway

P2Y receptors (P2YRs) are G protein-coupled receptors (GPCRs) with eight subunits (P2Y1, 2, 4, 6, 11–14) that respond to extracellular nucleotides, in contrast to P2X receptors [60]. An increasing amount of experimental evidence suggests that P2YRs play a significant role in tumor progression and metastasis [21]. P2YR functions and roles in gastrointestinal and prostate cancer have been studied but remain unclear [32, 61]. These studies appoint a possible new target in prostate cancer. P2Y1 receptors usually inhibit cancer cell proliferation, whereas P2Y2 receptors promote it [62]. As a result, we hypothesize that inhibiting or activating these receptors may affect prostate cancer treatment and prognosis (Fig. 2).

### P2YR in prostate cancer cell growth

Researchers have extensively investigated P2YRs in their role in cell death and survival. Shabbir et al. studied the purinergic receptor–mediated apoptosis in CRPC [49]. Furthermore, prostate cancer cells express a functional P2-purinergic receptor linked to phospholipase C, and agonists of this receptor significantly inhibit the proliferation [47]. P2Y2 triggered Src, which phosphorylated p38, resulting in COX-2 overexpression and apoptosis resistance in both colorectal HT-29 and prostate DU145 cancer cells [63]. Indeed, ursolic acid increased intracellular ATP levels, likely released into the extracellular area to activate P2Y2 [64, 65]. This apoptosis resistance mechanism is critical for immune-targeted treatment in prostate cancer.

In contrast, using a selective P2Y1 receptor agonist, ADP analogue MRS2365, activation of P2Y1 receptor–induced cell death suppressed the proliferation of PC-3 cells [66]. They illustrate P2Y1 receptor’s ability to trigger ERK1/2 phosphorylation, which regulates several cellular processes such as cell membrane potential, proliferation, and programmed cell death [60]. P2X7 is also known to promote ERK1/2 activation, which causes the death of prostate cancer cells. This coincidence could provide us with further information about the role of P2 receptors in regulating cell proliferation. A study of the interaction and dynamics of P2Y1 receptors with their novel ligands by structure-based computing and docking analysis led to the same conclusion that P2Y1R activation induced apoptosis via the caspase 3/7 and reactive oxygen species (ROS) signalling pathways [67]. Therefore, P2Y1 receptor merged as a potential target for prostate cancer treatment [68].

P2YR also inhibited the proliferation of prostate cancer cells. The identical P2Y2 and P2Y11 proteins are also found in prostate cancer cells [69]. Therefore, it can effectively suppress prostate cancer progression in the androgen-sensitive, androgen-independent, and bone adaption phases [48].

### P2YR in prostate cell invasion and metastasis

Previous studies found that ATP increased the invasion and migration of prostate cancer cells via P2YRs [70]. Both in vitro and in vivo ATP-promoted invasion and migration of prostate cancer cells were significantly inhibited after P2Y2 receptor knockdown, indicating a role of the P2Y2 receptor in prostate cancer cell invasion and migration [71]. Furthermore, the P2YR receptor triggered ATP-induced phosphorylation of EGFR and ERK1/2, resulting in mouse oral cancer cell invasion and migration [60, 72]. ATP influences IL-8, Snail, E-cadherin, and claudin-1 via downstream ATP treatment related to EMT and metastasis-related genes [71]. Increased IL-8 and Snail expression lowered E-cadherin and claudin-1 expression [17]. The derived IL-8, on the other hand, promotes tumor migration in a variety of malignancies, such as cervical and prostate cancer. ATP and UTP significantly increased the expression effect, which was reduced in P2Y2-silenced cells and suppressed further following P2Y2 knockdown, demonstrating that the P2Y2 receptor was necessary for ATP-mediated expression of EMT/invasion-related genes in prostate cancer cells [71].

### The clinical implication of P2YR in prostate cancer

There is currently no clinical study on P2YRs associated with prostate cancer. P2Y12 receptor–selective antagonists, such as clopidogrel, have been identified to modulate the positive benefits of platelet function in cancer due to the critical role of P2YRs in other malignancies [73, 74]. Classic combination treatments, including chemotherapeutics and clopidogrel combined therapy, have shown promise in managing gastrointestinal cancer [73]. Meanwhile, by exposing cervical cancer cells to extracellular ATP, P2Y receptors can be selectively induced to absorb and accumulate in ionic cytotoxins [75]. This evidence suggests that this strategy could be utilized to deliver medications selectively to cancer cells rather than normal ones, giving anticancer medicines a novel twist. However, some advancements in research for these cancers have been made. They discovered that P2YRs could be essential targets for site-specific delivery in these cancers.

## Concluding remarks

Purinergic receptors have been extensively studied in various cancers. We have highlighted the significance of P2X and P2Y receptors in prostate cancer in this review. These purinergic receptors play critical functions in the prostate cancer microenvironment (Tables 1 and 2). In terms of clinical application, P2X7 can employ statins to inhibit the aggressive proliferation of PTEN-positive cells in prostate cancers induced by extracellular ATP. Furthermore, P2X7 can aid in the treatment and early detection of prostate cancer. P2X4 is another purinoceptor that could be used as a therapeutic target.

**Table 1:** The role of different purinoceptors in prostate cancer

	Purinergic receptors		Function	Feedback	Method
The molecular mechanism of purinoceptors acting on prostate cancer	P2XR	P2X7	Prostate cancer cell growth	Stimulation	MAPKs/p38 & ERK1/2/PI3K/AKT/mTOR [37–39]
		P2X7		Stimulation	HIF-1α/VEGF [35, 44]
		P2X4		Stimulation	PTEN/PI3K/AKT [45]
		P2X5		Counteract	NA
		P2X7	Prostate cancer cell invasiveness	Stimulation	HIF-1α/AKT/ERK1/2 [51]
				Stimulation	HIF-1α/MMPs [55, 56]
	P2YR	P2Y2	Prostate cancer cell growth	Stimulation	Src/p38/COX-2 [63]
		P2Y1		Counteract	ERK1/2 [60] & ROS/Caspase 3/7 [67]
		P2Y2 & P2Y11		Counteract	NA
		P2Y2	Prostate cancer cell invasiveness and metastasis	Stimulation	EGFR/ERK1/2 [60, 72]

**Table 2:** The role of different purinergic receptors in the clinical significance of prostate cancer

	Purinergic receptors		Function	Method
The clinical significance of purinergic receptors in prostate cancer	P2XR	P2X7	Statins modulates invasiveness [52]	Downregulate the nuclear level of phosphorylated AKT through P2X7 [52]
		P2X7	Diagnose the early stage of prostate cancer [55]	This stage-specific labeling pattern has been classified into three purinergic receptor translocation categories (PRT0-3) [55]
	P2YR	NA	NA	NA

On the one hand, P2XR plays an essential role in promoting prostate cancer cell growth/inhibition and invasiveness/metastasis. P2YR, on the other hand, may promote the proliferation and metastasis of prostate cancer cells via various mechanisms. Understanding the purinergic signal in prostate cancer cells promises future prostate cancer research, both transformational and mechanistic.

## Data Availability

Not applicable.
